# Remarks on the General Treatment of Strangulated Hernia, with a Report of Two Cases

**Published:** 1861

**Authors:** A. Fisher

**Affiliations:** Chicago


THE CHICAGO
MEDICAL EXAMINER

VOL. II. APRIL, 1861. No. 4

### ORIGINAL CONTRIBUTIONS.

*Remarks on the General Treatment of Strangulated Hernia, with the report of two cases.* By A. FISHER, M. D., *Chicago. Read to the Chicago Medical Society.*

The principal indication in the treatment of strangulated hernia, is the reduction of the protruding viscera, or whatever the hernial sac may contain.

In order to accomplish our purpose successfully, it is necessary to understand as clearly as possible, the condition of the parts concerned in forming the hernial tumor, in the particular case.

We should ascertain whether the hernia is recent or of long standing, whether it has usually been reduced without difficulty, if indurated and painful, how long it has been so, also the state of the system, pulse, &c. By obtaining a perfect history of the case, we are enabled to proceed more jndiciously in the treatment of it, and we can judge better how long it will be safe to continue our manipulations for reduction, before resorting to an operation.

Should there be no induration or tenderness of the hernial tumor, we can make a trial for reduction at once; by placing the patient in the best position for relaxing the abdominal muscles as much as possible. Then by carefully manipulating the neck of the tumor with one hand, whilst steadily and firmly pressing with the other, in accordance with the case, we may sometimes accomplish our object. But without previous preparation we are often foiled; if so, we should loose no time in properly preparing the patient for a thorough trial.

In a majority of the cases, cold applications to the tumor will be of great service in relieving the pain and preparing the parts for reduction. For that purpose, I have been in the practice of applying ether, so as to keep up a constant evaporation, and have been much pleased with its effects; usually in less than an hour after applying it, the tenderness and pain subsides, cold produced by pounded ice may be equally beneficial, but I have succeeded so well with ether, that I have never used it. By whatever manner we make use of cold, it acts beneficially by condensing the gases, and constringing the vessels, thereby preventing or subduing congestion, and inflammation of the parts, though in cases where the constitution is feeble, the circulation languid, and gangrene is feared, it is not always safe to apply it. As a general rule, when cold applications are uncomfortable to the patient, and they do not lessen the pain and tumefaction, they should be discontinued, and warm or hot fomentations used in their stead, but the change should not be sudden, for reasons too obvious to mention.

In a practical point of view it is well to recollect that strangulation generally occurs, either by actual constriction of the protruding parts, producing inflammation, congestion and swelling, and consequently strangulation; or the inflammatory process may first commence in the viscera contained in the hernial sac, causing the parts to swell, thereby producing strangulation by pressure on the walls of the opening.

In the former case our resources are limited, for it is difficult to reduce the inflammation when we cannot relieve the stricture that caused it, but in the latter, if we can reduce the inflammation we remove the cause, and reduction is generally easily accomplished.

Should we become satisfied that strangulation was caused by the stricture, it would be necessary to resort to an operation earlier than otherwise, for the prospect of success by taxis in such cases is small.

Complete anesthesia is undoubtedly beneficial in relaxing the system, when there is no inflammatory action. But when the pulse is frequent, hard and wiry, with evident signs of active inflammation by whatever manner caused, the tincture of veratrum viride given in full doses, is without doubt a remedy of great value, with it we can control the circulation and completely relax the system, consequently prevent or relieve the inflammatory condition, and bring the parts into the best possible state for reduction. I have never had occasion to try the veratrum in cases of this kind, but from its modus operandi, I believe that we can accomplish with it, all, or more than we can by bleeding, emetic tartar, or tobacco injection, and the system will be left in a much better condition when the effects of the remedy pass off, than it would be after using the remedies above named.

Opium or morphine in full doses, in conjunction with, or following the administration of the tincture of veratrum viride, is without doubt a valuable remedy to allay pain and irritation and restore the circulation to its normal standard.

When the system is properly prepared for reduction, we should loose no time in making judicious taxis, according to the indications in the particular case, we should always be sure that we are making pressure in the right direction, and that we do not injure, the parts, by continuing our efforts too long without intermission. If proper treatment has not been neglected until organic lesion has commenced, reduction can generally be accomplished. Though there are cases which cannot be reduced without an operation, by any art or skill, even when there are no adhesions. A case of the kind I once witnessed, and will here relate it.

The patient, a man about sixty-five years old, of German descent, had been troubled with a large scrotal hernia for more than twenty years, little or no attention was paid to it, generally it would come down during the day and return at night; finally it remained in the scrotum most of the time. After a while it began to be painful and the patient tried numerous times to reduce it, but failed; he then sent for his son, who was a well educated physician, and he made judicious efforts to return it at different times, for two or three days, but did not succeed, and as inflammatory symptoms began to supervene, he sent for me to operate. I found a scrotal hernia on the right side, as large or larger than my fist, it was not very tender on pressure and the case was quite chronic, and as the constitutional symptoms were not urgent, I proposed a trial for reduction before operating; the doctor thought it useless, but consented that I should make a trial. My efforts at reduction being unsuccessful, we proceeded to operate, by making a longitudinal incision through the integuments the length of the tumor. I then carefully dissected down to the sac and divided the stricture exterior to it, and made efforts for reduction without opening it, but did not succeed; upon laying open the sac, I found a number of coils of the intestines protruding which were a good deal congested, though there were no adhesions or signs of gangrene, but they were much distended with gas and their contents. The coils of intestine were so twisted and tangled at the neck of the tumor, and the parts were so swollen, that it was very difficult to return them, even when we could see just how to manipulate. It was only with great care and perseverance that we accomplished the reduction, although the opening was large. In this case the strangulation was occasioned principally, if not entirely, by the twisting of the neck of the hernia upon itself, perhaps caused, or at least made worse, by the long continued efforts at reduction.

The patient was comfortable after the operation, the wound healed kindly, and the operation was successful.

We learn by this case that hernia is not always reducable without an operation, when the opening is large and there are no adhesions, also the necessity of being guarded in our prognosis in all cases, for in fact we are never sure of reduction, until it is accomplished.

Should the taxis succeed, the patient ought to remain in a recumbent position and be kept perfectly quiet until all inflammatory symptoms have subsided, and a truss or bandage should be properly applied to prevent a return of the difficulty. The bowels should not be disturbed by cathartics for a number of hours after reduction, for that portion included in the stricture usually remains partially paralyzed for some time after it has been returned, impairing the peristaltic motion, so that the contents of the bowels above might be arrested at that point, and produce inflammation. When the taxis with all our auxiliary means have been fairly tried without success, we should proceed unhesitatingly to operate immediately, and not defer, and hope against reason and common sense, until the time for a successful operation has passed. Surgeons generally agree that error is more frequent by delay, than precipitancy, however that may be, it is certain that many persons loose their lives by strangulated hernia, that in all probability might have been saved by a timely operation.

Occasionally we are not called to see cases of strangulated hernia until the time for any attempt at reduction has passed, and gangrene sloughing, or other organic lesion has already taken place. What are we to do in such cases?

I would say by all means operate, unless the patient is in the last stages of collapse, for without an operation death will surely be the result, and by it we may perhaps prolong life for a short time, with the possibility of making a permanent cure, especially in chronic cases.

In illustration of the recuperative powers of nature, when properly assisted, and to show the propriety of operating in dubious cases of strangulated hernia, I will read the notes of an interesting case which occurred in my practice a number of years since, as it has never been published.

Mrs. Shoub, a German lady, about 60 years old, of a bilious and nervous temperament, with a good constitution, had been afflicted with femoral hernia for about fifteen years, but had never suffered much inconvenience from it until about a week before I saw her, which was December 28th, 1841.

For the previous week she had been treated by a homœopathist, who not understanding her case, tried to move her bowels by little pills, and not succeeding, gave a full dose of calomel and jalap, which of course only added to her suffering, without producing the desired effect.

Upon examination I found a tumor in the left groin about the size of a hens egg, colour natural, but quite tender on pressure, could feel indistinct fluctuation. I made no effort to reduce it, for the reason that it had not been returned for the past year, and fearing from the constitutional symptoms that much pressure might rupture the protruding intestine. Her tongue was dry and red, pulse 130 small and week, extremeties cold and clamy, nausea with occassional efforts to vomit, urine dark and small in quantity, bowels somewhat disturbed and very tender on pressnre, particularly in the iliac region; finally she had every sign of gangrene, or sloughing of the hernia.

It was then about 2 o'clock in the morning, and as I resided about 6 miles from the patient, I prescribed a full dose of morphine to be repeated in 3 or 4 hours and left her, stating to her husband and friends the nature of her case, that nothing but an operation would save her, and I feared it too late even for that to be successful, but told them if she was no worse by day light, that I would operate, if they wished me to.

I was informed early in the morning, that she was better, and requested to see her and operate if necessary. I immediately left, taking with me my partner Dr. Warner, and invited Dr. Armstrong, whose residence was on our way to assist us. We carefully examined the patient, and found no material alteration in her condition since I left her. I again stated to the patient, and her husband, the probable result of an operation, viz: that she might die in spite of the operation, and if she did survive it and recover, the contents of the bowels might pass out of the wound, and that she would then have to suffer the terrible affliction of an artificial anus, but that death in a short time would surely be the result, without it. Notwithstanding our unfavorable prognosis, they consented to the operation.

After placing the patient in a proper position, and getting in readiness, I commenced the operation, by making a longitudinal incision through the integuments about 4 inches long, then carefully dissected down to the sac, which was thickened and contained about 2 ounces of dark colored serum. The hernia consisted of a portion of the small intestine, protruding about an inch and a half through the opening. It was of a very dark purple color, and appeared to be thickened and softened, with strong adhesions of long standing.

I carefully introduced the point of my finger on which I guided my bistory and divided the strictures, and the severe pain was at once relieved.

We made no attempt to return the intestine but drew the edges of the wound together partially, though not so close, as to prevent the passage outwards of the fœces should the intestine slough which we were confident it would do in a short time. The patient was much relived by the operation, but then even evident signs of peritonitis still remaining, indicating alterative doses of calomel combined with opium, which were given, with the application of mercurial ointment externally. The next day, 24 hours after the operation, I saw her again, and just before my arrival the protruding portion of intestine gave way, and the contents ot the bowels above passed freely through the opening, giving great relief to the patient, pulse 120, tongue more moist, bowels less tender; continued nearly the same treatment for 3 or 4 days, when a slight mercurial impression was obtained, and the inflammatory symptoms pretty much subsided. I then made a thorough examination of the parts, and found that the protruding intes tine had entirely sloughed away, that the intestines were firmly agglutinated together and to the walls of the abdomen, completely preventing the fæcal matter from passing into the peritoneal cavity, I could see the contents of the bowels above passing out of the intestine, I finally discovered another opening into the lower portion. I then drew the wound together closely with adhesive straps, in hopes by doing so, to get a passage through the entire intestinal canal, but left directions to remove the straps, and let the accumulations pass out, should there be much pain and pressure. The plasters had to be removed once only, and in the course of two or three weeks from the time of the operation, the passage was completely established without any obstruction.

We then carefully pared the edges of the wound, and brought them together and confined them with sutures and adhesive plaster, giving opiates at the same time, to keep the bowels quiet, thereby preventing accumulations from interfering with the healing process. Nothing passed from the wound, after it was brought together, and in 3 or 4 days it was entirely sound, and she recovered rapidly without further treatment.

I saw the patient about ten years afterwards, her health had been good ever since the operation, she had never had any soreness or difficulty with the wound, or taken a dose of medicine, she was then nearly 70 years old.

The happy result of the above case, was quite unexpected, after I found that the adhesion prevented the contents of the bowels from passing into the peritoneal cavity, my highest aim was to prolong the life of the patient by an artificial anus. I might even say that after the passage was fully established, and the wound completely healed, I did not expect the cure would be permanent, for I still feared that the fæces would collect in the space intervening between the upper and lower portions of the intestines, producing pain, irritation, and ulceration, forming an abscess consequently establishing a permanent opening.

I have said nothing, as you will perceive respecting the manner of proceeding in the reduction of the different varietes of strangulated hernia, or on the various modes of operating in particular cases, for it was not my intention, my principle object being to show the necessity of proceeding with energy, carefully watching the patient, so as to be in readiness to take the advantage of the best opportunity for reduction, and then should our efforts be unavailing, to be prepared to operate in time to save the patient.

